# Typical structure of rRNA coding genes in diplonemids points to two independent origins of the bizarre rDNA structures of euglenozoans

**DOI:** 10.1186/s12862-022-02014-9

**Published:** 2022-05-09

**Authors:** Paweł Hałakuc, Anna Karnkowska, Rafał Milanowski

**Affiliations:** Institute of Evolutionary Biology, Faculty of Biology, Biological and Chemical Research Centre, University of Warsaw, Żwirki i Wigury 101, 02-089 Warsaw, Poland

**Keywords:** rRNA, rDNA, rRNA operon, Euglenozoa, Diplonemids, Euglenids, Kinetoplastids, Internal transcribed spacer

## Abstract

**Background:**

Members of Euglenozoa (Discoba) are known for unorthodox rDNA organization. In Euglenida rDNA is located on extrachromosomal circular DNA. In Kinetoplastea and Euglenida the core of the large ribosomal subunit, typically formed by the 28S rRNA, consists of several smaller rRNAs. They are the result of the presence of additional internal transcribed spacers (ITSs) in the rDNA. Diplonemea is the third of the main groups of Euglenozoa and its members are known to be among the most abundant and diverse protists in the oceans. Despite that, the rRNA of only one diplonemid species, *Diplonema papillatum*, has been examined so far and found to exhibit continuous 28S rRNA. Currently, the rDNA organization has not been researched for any diplonemid. Herein we investigate the structure of rRNA genes in classical (Diplonemidae) and deep-sea diplonemids (Eupelagonemidae), representing the majority of known diplonemid diversity. The results fill the gap in knowledge about diplonemid rDNA and allow better understanding of the evolution of the fragmented structure of the rDNA in Euglenozoa.

**Results:**

We used available genomic (culture and single-cell) sequencing data to assemble complete or almost complete rRNA operons for three classical and six deep-sea diplonemids. The rDNA sequences acquired for several euglenids and kinetoplastids were used to provide the background for the analysis. In all nine diplonemids, 28S rRNA seems to be contiguous, with no additional ITSs detected. Similarly, no additional ITSs were detected in basal prokinetoplastids. However, we identified five additional ITSs in the 28S rRNA of all analysed metakinetoplastids, and up to twelve in euglenids. Only three of these share positions, and they cannot be traced back to their common ancestor.

**Conclusions:**

Presented results indicate that independent origin of additional ITSs in euglenids and kinetoplastids seems to be the most likely. The reason for such unmatched fragmentation remains unknown, but for some reason euglenozoan ribosomes appear to be prone to 28S rRNA fragmentation.

**Supplementary Information:**

The online version contains supplementary material available at 10.1186/s12862-022-02014-9.

## Background

Until a few years ago, Diplonemea (Euglenozoa, Discoba) was a rather neglected group. Only two diplonemid genera, *Diplonema* and *Rhynchopus,* have been described and cultured. This remained in sharp contrast to other well-studied euglenozoans: Kinetoplastea, sister to Diplonemea, and Euglenida [1]. Lately, metabarcoding surveys from the deep pelagic zone and deep-sea sediments have shown their unrivaled diversity [2–4]. Based on these metabarcoding data, two clades of deep-sea pelagic diplonemids (DSPD I and II) have been described, with the former grouping 97% of all known diplonemid diversity [1]. It also encompasses ten diplonemid single-cells for which genomes were acquired [5]. The majority of the metabarcoding sequences corresponded with a single cell known as Cell 37, and were later described as a new species *Eupelagonema oceanica* [6].

The genomes originating from single cells were incomplete and fragmented, primarily due to high repetitiveness caused by an unexpectedly high density of ‘noncanonical’ introns, similar to euglenid nonconventional introns [5, 7]. The second reason is the size of the genomes – acquired assemblies were up to 300 Mbp large, consistent with the previously reported expected genome size of diplonemids [8]. However, even for such incomplete assemblies, regions present in many copies—such as mitochondrial DNA or nuclear ribosomal RNA (rRNA) operon—can be extracted [9, 10].

Typically, eukaryotic ribosomes contain four rRNAs. Three of them: 18S (also known as SSU, small subunit), 5.8S and 28S rRNA (together also known as LSU, large subunit) are encoded in a single operon (rRNA or rDNA) and co-transcribed. Genes are separated by internal transcribed spacers (ITSs), which are removed during post-transcriptional processing to form mature rRNAs [11]. Such a structure of four continuous rRNAs has been confirmed in the single investigated diplonemid *Diplonema papillatum* [12]. However, this result is in opposition to two other euglenozoan groups: kinetoplastids and euglenids. In both of these groups, 28S rRNA is fragmented into several smaller molecules: 6 in kinetoplastids [13–15], and 13 in euglenids [16–18]. These smaller rRNAs together perform structural and catalytic functions of typical 28S rRNA. The fragmentation is caused by additional ITSs in the rRNA operon of both euglenids and kinetoplastids. While 28S rRNA fragmentation occasionally occurs in various eukaryotes [19–21], the extent of the fragmentation in Euglenozoa is unparalleled. The lack of studied rRNA operons in diplonemids puts the parsimonious (i.e., involving single ancestral acquisition) evolutionary path of euglenozoan rRNA operon into question, which we try to answer herein.

## Results

We successfully assembled rRNA operons of all three classical and six out of ten deep-sea diplonemids, including the most abundant *Eup. oceanica* (Additional file 2: Table S1). Lengths of all acquired operons and their subunits are typical for eukaryotes. Furthermore, we acquired and annotated sequences of rRNA operons for three euglenids—one heterotrophic species and two phototrophs—and for nine kinetoplastids—six metakinetoplastids and three prokinetoplastids. In several cases a complete intergenic region (IGR) has not been recovered, hence only the 18S-5.8S-28S rRNA coding region has been analysed further.

Since it is not possible to automatically predict the very complex rRNA secondary structure, another approach has been utilised. We used previously described rRNA structures of *Euglena gracilis* [17], *Trypanosoma cruzi* [22] and *Leishmenia major* [23] to identify structural elements, i.e., helices and loops composing the bulk structure of the ribosome. Subsequently, we modelled these structural elements for all other species (see “Methods” section) and marked them upon the alignment. The expected structure of the mature rRNA, which is the most conserved known biological feature, has been used to identify expansion elements. The fragments which would disrupt the ribosome structure are most likely removed during the maturation of rRNA. All alignments and annotations are available in the RepOD repository accompanying this paper (https://doi.org/10.18150/J4Q2ES).

**Fig. 1.**
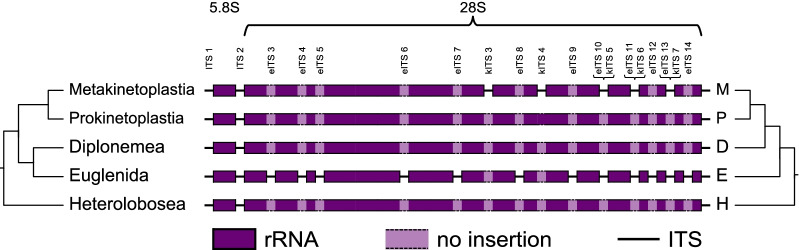
Schematic distribution of identified internal transcribed spacers in the LSU rDNA of euglenozoans. The 5.8S and 28S rRNA gene structure has been shown for Diplonemea, Euglenida, two main clades of Kinetoplastea, and Heterolobosea as an outgroup. Additional ITSs have been numbered within euglenids (eITS) and kinetoplastids (kITS), with ITSs present in homologous positions marked (eITS10/kITS5, eITS11/kITS6 and eITS13/kITS7). On the left, phylogeny of Euglenozoa has been shown. For comparison, phylogeny presented in Kostygov et al*.* (2021) has been shown on the right. In both cases additional ITSs cannot be traced to the common ancestor, indicating their independent origin

We identified all conserved features of the 28S rRNA in all analysed diplonemids and no significant insertions or deletions were found (Additional file 2: Table S1, Fig. 1). For that reason, we conclude that no additional ITS is present in diplonemid rRNA operons, resulting in the typical eukaryotic continuous 28S rRNA.

On the other hand, the rRNA operons of all three euglenids are significantly elongated (10–13 kbp), mainly due to the large expansions within 18S and especially 28S rRNA genes (Additional file 2: Table S1). As previously suggested, almost all of the expansions occur in divergent regions of the rRNA, also known as expansion segments (ES) [24, 25]. We identified the ITSs described for *E. gracilis* [26] in both analysed euglenids. Moreover, two potentially novel additional ITS sites were found in *Rhabdomonas* *costata*. All expansions within 18S rRNA are shared between euglenids but it has been shown in *E. gracilis* that they are not removed from the mature 18S rRNA [16]; hence we do not indicate these as additional ITSs.

In kinetoplastids, two types of the rRNA operons can be distinguished: an elongated one in metakinetoplastids (trypanosomatids and bodonids), and a standard eukaryotic one in all prokinetoplastids (*Perkinsela* sp. and similar). Long rRNA operons in metakinetoplastids originated from the elongations of the 28S rRNA gene, which are present in the same positions and possess the same features and spatial distribution pattern as previously described additional ITSs [13–15]. All analysed metakinetoplastid sequences have exactly the same pattern of additional ITSs as trypanosomatids (Fig. 1). In *Bodo* *saltans*, a basal metakinetoplastid, expansion in the kinetoplastid ITS3 (kITS3) site is short but still much longer than in prokinetoplastids and other analysed species. It suggests that *B.* *saltans* rRNA does contain the kinetoplastid ITS3. Furthermore, only three kinetoplastid ITSs share positions with euglenid ITSs: kITS5 and eITS10, kITS6 and eITS11, kITS7 and eITS13 (Fig. 1, Additional file 1: Figure S1).

No expansions were recognised as group I introns by RNAmmer [27]. No homology has been observed within and between additional ITSs of euglenids and kinetoplastids. The sequences of ITSs do not have distinct or conserved secondary structures and we did not recover any open reading frames (ORFs) longer than 20 amino acids. No significant blast hits (e-values < 0.001) to NCBI-nr and NCBI-nt have been recovered.

**Fig. 2.**
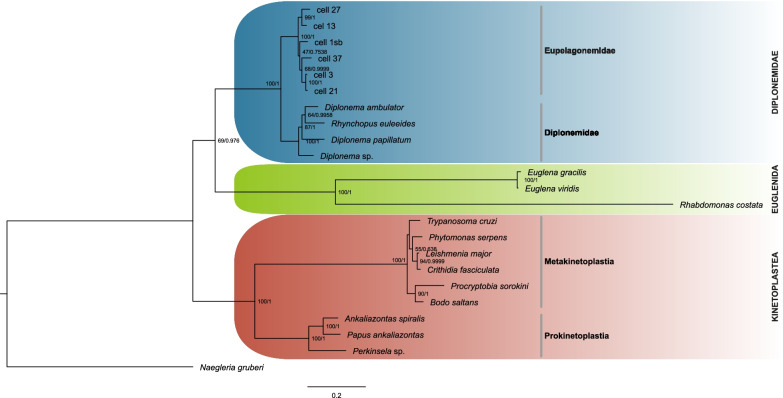
Maximum likelihood tree of Euglenozoa based on 4817 nucleotide positions of the rRNA operon. Bayesian inference resulted in the same topology. ML bootstrap (BT) and Bayesian posterior probability (pp) values are indicated at the nodes (BT/pp)

To provide background for structural analyses we reconstructed the phylogeny of Euglenozoa based on the 18S-5.8S-28S rRNA coding region (Fig. 2). All three major groups of euglenozoans form maximally supported clades (100 bootstrap for ML and 1.00 posterior probability for BI). In spite of that, relations between groups are not resolved, though this is typical for rRNA phylogenies of Euglenozoa [9, 28, 29]. The internal topology for euglenids, kinetoplastids and diplonemids is as expected [1], moreover, within diplonemids, the division between Diplonemidae and Eupelagonemidae is maximally supported, and the internal topologies of these families are in agreement with the previous analysis [6].

## Discussion

The typical eukaryotic 18S-28S rDNA unit comprises co-transcribed 18S, 5.8S and 28S rRNA separated by ITS1 and ITS2, which are removed in post-transcriptional processing. The ITS2 is a eukaryotic invention—the 23S rRNA present in prokaryotes comprises both 5.8S and 28S rRNA structure, and is separated from 16S rRNA (the prokaryotic equivalent of 18S) by a single ITS. The length and secondary structure of the ITSs are not conserved, with the shortest ITSs observed in the protist parasite *Giardia intestinalis,* and the longest—in multicellular eukaryotes [11, 30]. The elongation is usually a result of insertion of short tandem repeats, but the functional consequence of such elongations is unknown.

Fragments of the rRNA (both 18S and 28S) forming external (more distant from the site of peptidyl transfer) parts of the ribosome are much less conserved than the internal fragments. For this reason, externally located variable regions (or expansion segments, ES) show much greater variability in sequence, structure and length [25]. Expansion of these segments causes the size of mature 28S rRNA to vary from ~ 2500 bp in microsporidia to over 5000 bp in multicellular species, such as humans. Interestingly, the LSU rRNA of microsporidia is a fusion of 5.8S and 28S rRNA, with a structure more similar to prokaryotes than other eukaryotes [31]. In several distinct eukaryotic lineages an opposite process occurred, resulting in the formation of a fragmented mature 28S rRNA. The best-known example is the presence of the so-called “hidden break” in insects and other protostome animals, causing the RNA isolates to seem to be degraded [19, 32, 33]. An analogous situation is observed in several mammals, mainly rodents [20, 34, 35]. It is worth mentioning that insect and mammalian “hidden breaks”, or rather additional ITSs, are present in different expansions’ segments (ES19 and ES15, respectively). Furthermore, in the case of the rodent *Ctenomys,* the additional ITS is present within an intron. Said intron is excised or retained in a tissue-specific fashion, resulting in the absence or presence of the “hidden break”, leading to continuous or fragmented mature 28S rRNA [20]. Different ribosome structures in different tissues may suggest the functional importance of the additional ITS in *Ctenomys*. Another notable example exists in malaria-causing apicomplexan *Plasmodium falciparum*, in which two types of 28S rRNA units are present: continuous A-type and fragmented S-type [36]. The expression of one or the other type is strictly regulated (e.g., by temperature and glucose concentration), with only the continuous A-type expressed in the vertebrate host [21, 37, 38]. Other non-homologous additional ITSs can be found in Amoebozoa [39], dinoflagellates [40] and in mitochondria or plastid 23-28S rRNA [25]. However, the number of additional ITSs present in kinetoplastids and euglenids is unmatched in any other taxa.

Newly acquired rRNA structures of nine diplonemids show that the lack of additional ITSs in *D.* *papillatum* is, in fact, typical for the Diplonemea. This finding is significant for elusive taxa like diplonemids, known mostly from metabarcoding data. Continuous 28S rRNA allows the employment of third-generation sequencing (PacBio, MinION) in both DNA and RNA surveys [41–43].

However, such a result is a surprise from the evolutionary point of view. The presence of additional ITSs in both euglenids and kinetoplastids suggests that it may be another ancestral feature of Euglenozoa, especially since three of them share positions between groups [44]. In such a scenario, continuous 28S rRNA in *D.* *papillatum* could be coincidental – species-specific secondary losses of additional ITSs (the aforementioned “hidden breaks”) are known in insects [19]. Lack of additional ITSs in all diplonemids rules out this possibility. Similarly, the lack of additional ITSs in Prokinetoplastida indicates that the last common ancestor of kinetoplastids had a continuous 28S rRNA. In such a case, additional ITSs found in kinetoplastids could be common only for Trypanosomatidae and Bodonidae, but exact pinpointing of their origin requires additional surveys across kinetoplastids [1]. If additional ITSs are neither an ancestral feature of kinetoplastids nor present in diplonemids, they cannot be common in Euglenozoa.

Based on the obtained results, it seems that the kinetoplastids’ and euglenids’ additional ITSs emerged independently. However, it is highly unlikely that the occurrence of additional ITSs in such unparalleled numbers in two closely related groups is a coincidence. It seems most probable that some factor in euglenozoan biology makes fragmentation of the 28S rRNA more feasible than in other eukaryotes. One possible explanation is the ribosomal protein repertoire unique to this group. It has been shown that post-transcriptional removal of additional ITSs in *T. brucei* is guided by ribosomal proteins [45]. In general, kinetoplastid ribosomal proteins exhibit a number of unusual features interacting with unusual rRNA [22, 46, 47]. Even more oddities have been found in cryo-EM structures of the *E. gracilis* ribosome [48]. Small rRNAs termini colocalise, mostly in two focal points. Several ribosomal proteins exhibit unusual elongations interacting with the expansion segments of *E. gracilis* rRNA, and four novel *Euglena*-specific ribosomal proteins have been found, three of them interacting with unique LSU rRNA motifs/deletions. Furthermore, *E. gracilis* rRNA was found to bear the highest number of ribosomal post-transcriptional modifications reported to date [49]. The frequency of modifications is much higher in the LSU, correlating with a high level of rRNA fragmentation. Similarly, a number of unique RNA modifications have been found in the proximity of additional ITSs in *T.* *brucei* [50]. A group of such modifications appears late in the maturation of the ribosome, at the same stage as ITSs removal. In any case, the co-presence of RNA modifications, unusual ribosomal proteins and additional ITSs suggests close correlation. Answering the “chicken or egg” question about their origin will require additional data with a better phylogenetic representation of euglenids and diplonemids.

## Conclusions

We acquired novel complete rRNA operons for six kinetoplastids, two euglenids, three classical and six marine diplonemids. All analysed diplonemids lack additional ITSs known from other euglenozoans. Interestingly, while all investigated metakinetoplastids have the exact same pattern of ITSs as trypanosomes, the early branching prokinetoplastids do not possess any additional ITSs. These results suggest that additional ITSs in euglenids and kinetoplastids are of independent origins.

## Methods

Genome assemblies of classical and deep-sea diplonemids were accessed [5]. During the initial analysis of the original assemblies, we have found highly fragmented rRNA operons only. For that reason, raw reads for each species were obtained and reassembled (Additional file 2: Table S2). The quality of raw reads was evaluated using FastQC v0.11.5 [51] and trimmed in Trimmomatic v0.36 [52]. Processed reads were assembled using metaSPAdes v3.10.1 [53, 54]. Acquired assemblies were searched by BLASTn with rRNA sequences of *Diplonema papillatum* (KF633466-8) as queries. To exclude potential mitochondrial or contaminant rRNA operons and potential misassembles only high scoring hits (e-value < 10^–5^) with high coverage (> 5 × higher than genome average) were kept. We have found that the newly performed metaSPAdes assemblies contained rRNA operons of better quality, and therefore they were used for further analyses. Assembly graphs were manually inspected in Bandage [55] to identify potential misassembles. In such a case, contigs containing rRNA operons were manually corrected and replaced in the assemblies. Furthermore, the acquired operons were manually checked for mismatches since metaSPAdes does not support mismatch correction [54].

To provide phylogenetic background, we searched genomes of kinetoplastids and euglenids for rRNA operons by BLASTn. The operons of *E. gracilis* (M12677.1, X53361.2) and *Crithidia fasciculata* (Y00055.1) were used as queries for euglenids and kinetoplastids, respectively. Genome assemblies of *Perkinsela* sp. (LFNC01000001.1) and *Phytomonas serpens* (AIHY00000000.1) were accessed from GenBank [56, 57]. Raw reads were accessed (last time on 05/05/21) for *Euglena viridis* (SRR14099996) [58], *Rhabdomonas costata* [59], *B. saltans* (ERR036178) [60], *Papus ankaliazontas* (SRR13394431), *Ankaliazontas spiralis* and *Procryptobia sorokini* (cocultured, SRR13394430) [61]. These were processed in the same way as diplonemid assemblies. Lastly, the rRNA operon sequence of *Naegleria gruberi* (AB298288.1) was accessed as a non-euglenozoan outgroup.

Sequences of rRNA operons were aligned using MAFFT einsi [62]. The obtained alignment was further manually edited in Geneious v10.2.2 [63], based upon annotated secondary structures. The secondary structure of *E. gracilis* rRNA has been predicted [16, 17], while for several trypanosomatid ribosomes, cryo-electron microscopy structures have been obtained [23, 46, 64]. RNApdbee 2.0 web‑server [65] was used to extract secondary structures from available cryo-electron microscopy models. Secondary structures of *E. gracilis, T. cruzi* and *L. major* have been modelled, based on previously published structures. Determined helices were marked upon the alignment and numbered following a previously published structure of *Saccharomyces cerevisiae* rRNA structure [66]. Using this profile, structures of particular domains and regions have been predicted for all species using the RNAfold WebServer [67, 68]. Several intervals have been used in each case to best identify structural elements, i.e., helices and loops composing the bulk structure of the ribosome. Helices have been numbered in the same manner and marked upon sequences of all newly analysed species. Based on this annotation, homologous helices were manually aligned to prepare structure-based alignment which was used to identify irregularities in the lengths of the analysed structures.

All identified expansions have been investigated for possible homologies on sequence or structure level and checked for presence of open reading frames or other potentially coding fragments. Their sequences were searched by RNAmmer [27], BLASTn against NCBI-nt and BLASTx against NCBI-nr. The expansions occurring in sites of known additional ITSs in *E. gracilis* and *T. cruzi* were described as corresponding ITS. Unusually large (> 4 × longer than in other species) expansions found in other divergent regions were marked as a potential novel additional ITSs.

An alignment produced by MAFFT was used for phylogenetic analyses. Fragments with very high variance and no conserved secondary structure were manually removed (e.g., ITSs), with retained alignment trimmed using trimAL v1.2rev59 with –automated1 option [69]. The remaining 4817 positions were used for phylogenetic analyses. A maximum-likelihood tree (ML) was calculated using raxml-ng [70], with GTR + I + G4 model of substitution chosen by modeltest-ng [71]. The best tree was estimated using 20 different starting trees and 1000 bootstraps. Bayesian inference was performed in MrBayes v3.2.6 [72]. Two runs of a Markov Chain Monte Carlo were carried out with four chains (one cold and three heated), with GTR + I + G model of substitution, 10 million generations, trees sampled every 100 generations and the burn-in set to the first 25% of the sample.

## Supplementary Information

**Additional file 1. Figure S1.** Location of additional ITSs within secondary structure of LSU.

**Additional file 2. Table S1-2.** Identified segments of the rDNA and their lengths.  List of analysed species and source of data.

## Data Availability

All the datasets generated and/or analysed during the current study are available in the RepOD repository, https://doi.org/10.18150/J4Q2ES.
