# Supplementary material for: Typical structure of rRNA coding genes in diplonemids points to two independent origins of the bizarre rDNA structures of euglenozoans
Source: BMC Ecol Evol. 2022 May 9;22:59. doi: 10.1186/s12862-022-02014-9 (PMC9082867; doi:10.1186/s12862-022-02014-9)
Supplement: Supplementary file 1 — Additional file 1. Figure S1. Location of additional ITSs within secondary structure of LSU. [file 12862_2022_2014_MOESM1_ESM.pdf]

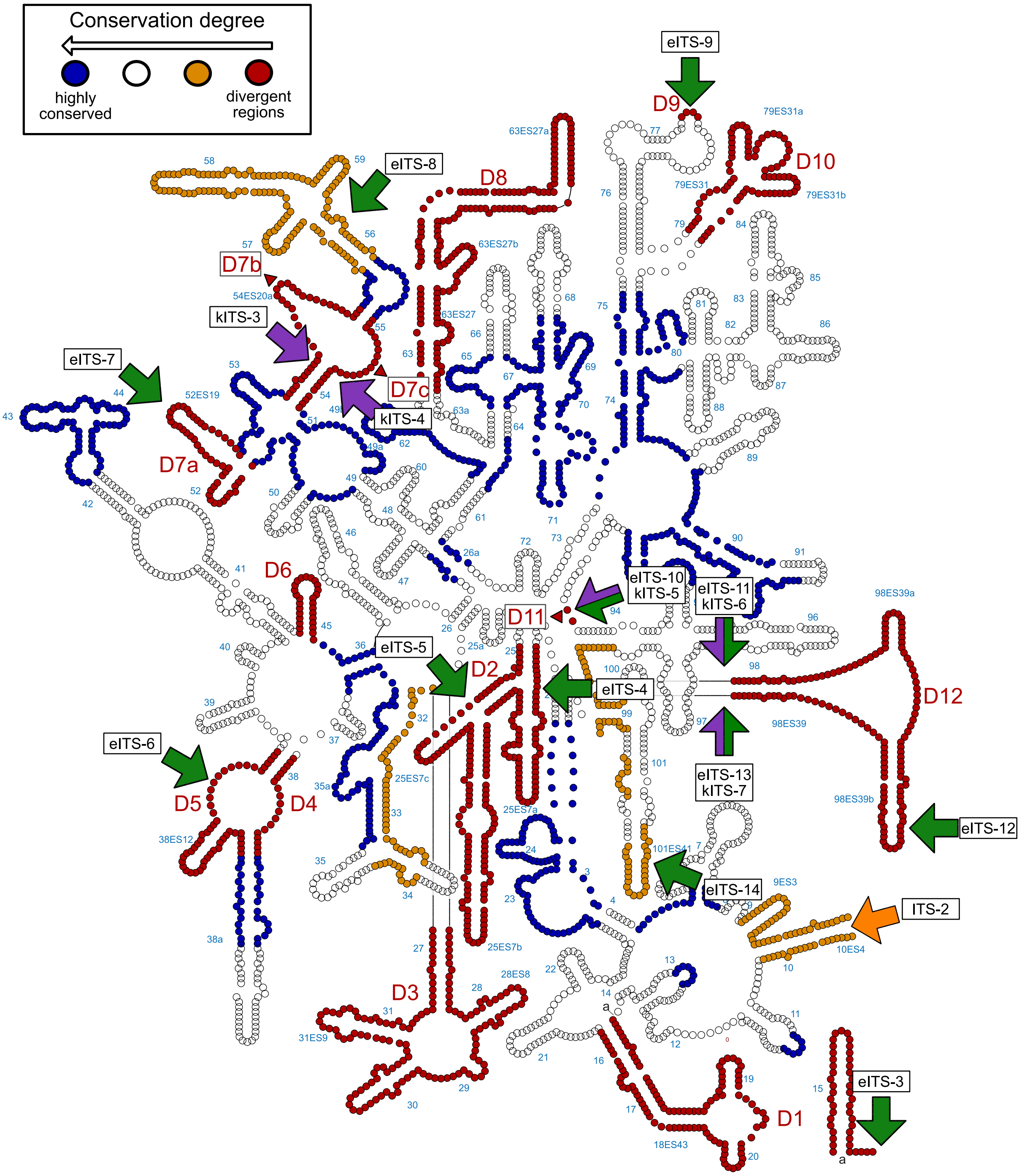

Supplementary Figure 1.

Location of additional ITSs within secondary structure of LSU.

The structure and helices' numbering (light blue) follows Bernier *et al.* 2014. Named divergent regions D1-D12 are shown in marked (following Hassouna *et al*. 1984). Arrows mark aITS sites and color denotes their occurrence: orange – ITS 2, green – euglenid aITSs, purple – kinetoplastid aITSs, green/purple – aITSs shared by euglenids and kinetoplastids.
